# A Comparative Study of the Second-Order Hydrophobic Moments for Globular Proteins: The Consensus Scale of Hydrophobicity and the CHARMM Partial Atomic Charges

**DOI:** 10.3390/ijms12128449

**Published:** 2011-11-29

**Authors:** Cheng-Fang Tsai, Kuei-Jen Lee

**Affiliations:** 1 Department of Biotechnology, Asia University, Taichung 413, Taiwan; E-Mail: tsaicf@asia.edu.tw; 2 Department of Bioinformatics, Asia University, Taichung 413, Taiwan

**Keywords:** hydrophobicity consensus scale, CHARMM partial atomic charges, Secondorder hydrophobic moment

## Abstract

In this paper, the second-order hydrophobic moment for fifteen globular proteins in 150 nonhomologous protein chains was performed in a comparative study involving two sets of hydrophobicity: one selected from the consensus scale and the other derived from the CHARMM partial atomic charges. These proteins were divided into three groups, based on their number of residues (*N*) and the asphericity (*δ*). Proteins in Group I were spherical and those in Groups II and III were prolate. The size of the proteins is represented by the mean radius of gyration (*R_g_* ), which follows the Flory scaling law, *R_g_* ∝ *N*^ν^. The mean value of *v* was 0.35, which is similar to a polymer chain in a poor solvent. The spatial distributions of the second-order moment for each of the proteins, obtained from the two sets of hydrophobicity, were compared using the Pearson correlation coefficient; the results reveal that there is a strong correlation between the two data sets for each protein structure when the CHARMM partial atomic charges, |*q_i_|* ≥ 0.3, assigned for polar atoms, are used. The locations at which these distributions vanish and approach a negative value are at approximately 50% of the percentage of solvent accessibility, indicating that there is a transition point from hydrophobic interior to hydrophilic exterior in the proteins. This may suggest that there is a position for the proteins to determine the residues at exposed sites beyond this range.

## 1. Introduction

Protein structures are stabilized by several non-covalent interactions, such as hydrophobic, van der Waals, electrostatic, and hydrogen bonding interactions [1,2]. Of all the interactions that take place in proteins, hydrophobic interaction is a dominant force that drives protein folding. The interaction between non-polar amino-acid residues and the aqueous environment provides a strong hydrophobic force for protein folding [1,3], forming a hydrophobic core in the protein interior. Thus, the variation in hydrophobicity from the hydrophobic core to hydrophilic exterior is the spatial variation for most globular proteins. To quantify the spatial transition of hydrophobicity between the interior and the surface of globular proteins of known structure, Silverman [4] introduced the second-order hydrophobic moment, using the hydrophobicity consensus scale of Eisenberg [5]. The second-order hydrophobic moment, or quadrupole moment, is similar to the electrostatic quadrupole, and it was first proposed by Eisenberg [5] to describe the hydrophobic distributions of proteins. A second-order hydrophobic moment has also been applied to the systems [6] including native and decoy protein structures, and multi-domain proteins [7]. In contrast, the first-order hydrophobic moment, or hydrophobic dipole moment, is similar to the dipole moment of a molecule. The first-order hydrophobic moment has been used to measure the amphiphilicity of primary and secondary structures either in globular proteins [5,8–10] or in transmembrane proteins [11,12]. Moreover, the zeroth-order hydrophobic moment is the total hydrophobicity of the amino-acid residues in a protein, and it is similar to a net molecular charge.

Residues classified as buried or exposed are conventionally described by a geometric parameter calculated using the solvent-accessible surface area (ASA) [13], which is generated by rolling a spherical probe with a radius of 1.4 Å over the surface of a protein. The ASA values obtained are in absolute values, and these can be changed to relative values, which are also known as the percentage of solvent accessibility *p* (%) [14,15]. These values have been calculated using the ASA value of each amino-acid residue in the native state normalized with respect to the ASA value of the corresponding residue X in the extended state either of Gly-X-Gly [16,17] or of Ala-X-Ala [14,15]. Recently, the prediction of solvent accessibility, based on protein sequences, has also been developed using support vector regression (SVR) [18–21]. Gromiha *et al*. [14,15] have classified these relative values to the locations of protein residues as follows: 0–2% as completely buried, 2–20% as a location between buried and partially buried, 20–50% as partially buried, and greater than 50% as completely exposed. This classification is used to study the stability changes for protein mutants.

Silverman [4] used an ellipsoidal representation for the shapes of proteins. The extent of an ellipsoid was defined by a distance, *d*, which was calculated from the molecular moments of geometry [4]. The second-order hydrophobic moment per residue in a protein was calculated from a small value of *d* to a larger value by increasing the value of *d* until all residues in the protein have been collected to obtain its spatial distribution of the second-order hydrophobic moment. In contrast, the percentage of solvent accessibility, *p* (%), is adopted in this work, and we employ successive increases of solvent accessibility; that is, increases from 0 to 100% are used to study the spatial distributions of the second-order hydrophobic moment of the protein structures. The second-order hydrophobic moment per residue is calculated from a space defined by a small value of *p* (%), which collected the residues at the hydrophobic core of a protein to the larger value of *p* (%) at which all residues will be collected. To investigate spatial characteristics, the distances from the origin of a protein to the centroids of the protein residues in a space defined by *p* (%) are also considered. Here, we use the center of mass of each protein as the origin. In this work, two sets of hydrophobic parameters for each of the amino-acid residues in the protein structures are used in a comparative study of the second-order hydrophobic moments: one is based on the hydrophobicity consensus scale of Eisenberg [5], and the other is derived from the partial atomic charges assigned to atoms of a protein in the CHARMM program [22].

In this work, a different approach from the works of Silverman [4] is used to define the spaces in a protein; *i.e.*, we employ *p* (%) to define the spaces to calculate the spatial distributions of the second-order hydrophobic moment of a protein. The purpose of this study is to examine whether the hydrophobicity of each residue in a protein can be obtained using molecular modeling, and whether the values of hydrophobicity derived from the partial atomic charges assigned to atoms of a protein in the CHARMM program [22] are comparable with those of the consensus scale of Eisenberg [5] by using the spatial distributions of the second-order hydrophobic moment in the new definition of spaces in the protein. This work may provide an alternative way to calculate the hydrophobicity of each residue in a protein. Since the hydrophobicity of an amino-acid residue cannot be defined and measured easily, it is usually obtained from the free energy changes calculated by transferring amino-acid side chains from aqueous to non-aqueous media [23,24] or from non-aqueous to aqueous media [25].

## 2. Materials and Methods

**Figure 1:**
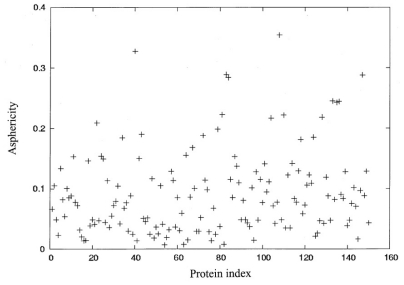
The asphericity (*δ*) as a function of protein indices.

A set of 150 nonhomologous protein chains was randomly selected from PDBSELECT [26], which included more than 4,000 protein chains. The sequence identity in this set of proteins is lower than 25%, and their single-chain protein crystal structures have been determined at a resolution of less than 1.8Å and at an R-factor of less than 0.18. All the structures of the proteins were obtained from the PDB [27]. A figure of asphericity (*δ*) and the index of 150 proteins are shown in Figure 1. In order to select protein structures with diverse sizes and shapes, fifteen structures, as shown in Table 1, were selected from these 150 proteins and divided into three groups, depending on the number of residues (*N*) and the extent of asphericity (*δ*).

The shape of a protein can be characterized in terms of eigenvalues of the radius of gyration tensor. The radius of gyration tensor *T* is defined as follows [28,29]:

(1) $$T=\frac{1}{n}\sum_{i=1}^{n}T_{i}T_{i}^{t}=\left[\begin{array}{ccc} t_{xx} & t_{xy} & t_{xz}\\ t_{yx} & t_{yy} & t_{yz}\\ t_{zx} & t_{zy} & t_{zz} \end{array}\right]$$

where *T_i_* = col (*x_i_, y_i_, z_i_*) is the vector going from the origin at the protein center of mass vector to the position of the centroid of residue *i*. The center of mass vector, **r***_CM_*, is defined by [4]

(2) $$r_{CM}=\frac{1}{N_{r}}\sum_{i=1}^{N_{r}}r_{i}$$

where *N_r_* is the number of residues in the protein, and **r***_i_* is the position vector for the centroids of the amino-acid residues. The matrix *T* can be diagonalized to obtain the three eigenvalues, α*_i_* (*i =* 1, 2, 3), of this tensor. The asphericity (*δ*) [28–31] has been used to characterize protein shapes, and δ is computed as

(3) $$\delta =1-3\langle \frac{\alpha_{1}\alpha_{2}+\alpha_{2}\alpha_{3}+\alpha_{1}\alpha_{3}}{{\left(\alpha_{1}+\alpha_{2}+\alpha_{3}\right)}^{2}}\rangle$$

If *δ* = 0, the shape is a perfect sphere, the extent of asphericity can be referred as 0 < *δ <* 1, and if *δ* = 1, the shape is a rod. The shape parameter (*S*) [30,31] has been used to quantify the whole shape of a protein, and *S* is computed as

(4) $$S=27\langle \frac{\prod_{i=1}^{3}\left(\alpha_{i}-\bar{\alpha }\right)}{{\left(\alpha_{1}+\alpha_{2}+\alpha_{3}\right)}^{3}}\rangle$$

Where

(5) $$\bar{\alpha }=\frac{\alpha_{1}+\alpha_{2}+\alpha_{3}}{3}$$

If *S = 0*, the shape is a perfect sphere, if *S < 0*, the shape is oblate ellipsoid, and if *S > 0* the shape is prolate ellipsoid. The shape of proteins can also be represented by semiaxes [32]; *i.e.*, *a* = [5(*α*_1_ +*α*_2_)/2]^1/2^ and *b* = (5*α*_3_)^1/2^, where *α*_i_ was sorted according to increasing magnitude. If *a* ≈ *b*, the shape is close to a sphere, and if *a* < *b* the shape is a prolate ellipsoid.

The molecular size of a protein can be probed using its mean square radius of gyration, 〈*R_g_*^2^〉, defined as follows [33]:

(6) $$\langle R_{g}^{2}\rangle =\frac{1}{N_{r}}\sum_{i=1}^{N_{r}}{\left(r_{i}-r_{CM}\right)}^{2}$$

where *N_r_*, **r***_i_*, and **r***_CM_* are defined in Equation (2).

The solvent accessible surface area (ASA) for each atom or for each residue in the proteins of interest was obtained using the computer program ASC [34] with default parameters. The percentage of solvent accessibility, *p* (%), was calculated as follows:

(7) $$p=\frac{{\mathrm{ASA}}_{X,folded}}{{\mathrm{ASA}}_{X,G-X-G}}$$

where *ASA_X,folded_* is the ASA of X residue in the folded state of a given protein and *ASA_X,G–X–G_* is the ASA of the corresponding residue X in tripeptide (Gly-X-Gly) with the extended state. The extended state (*ϕ* = *Ψ* = 180°) coordinates were generated using InsightII program [35]. The Gly residues to either side of interest residue X can provide steric screening effects of neighboring residues in the simulated models, and such steric screening effects can reduce the values of ASAs calculated for the X residues in the extended state. The results of the ASA for each residue type X are shown in Table 2

Polar atoms were defined on the basis of the partial charges, which were assigned to atoms in a protein by the CHARMM program [22]. In this work, three sets of partial charges, namely, |*q_i_*| ≥ 0.25, 0.27, or 0.3 were assigned as polar atoms, and the remaining atoms were considered apolar. These three sets of partial charges were used to examine which one has the best correlation with the hydrophobicity consensus scale of Eisenberg [5]. Polar and apolar ASAs of each residue were calculated by summing the ASA of the respective polar and apolar atoms in the residue. The hydrophobicity, *h_i_*, for each amino-acid residue, *i*, in a protein was calculated as follows:

(8) $$h_{i}=\frac{{\mathrm{ASA}}_{apo}-{\mathrm{ASA}}_{po}}{{\mathrm{ASA}}_{apo}+{\mathrm{ASA}}_{po}}$$

where *ASA_apo_* and *ASA_po_* are the apolar and polar ASAs, respectively, in the residue. For a given protein, the *h_i_* value of each residue, either selected from the hydrophobicity consensus scale of Eisenberg [5], as shown in Table 2, or calculated according to Equation (8) was normalized by the following equation:

(9) $$h_{i}^{\prime }=\frac{h_{i}-\bar{h}}{\sigma (h)}$$

where *h̄* is the mean of the *h_i_* for all residues in the protein, and σ(*h*) is their standard deviation. In this work, the second-order hydrophobic moment, *H*_2_(d*_i_*), per residue is similar to the works of Silverman [4], given by

(10) $$H_{2}(d_{i})=\frac{1}{n_{p}}\sum_{i}h_{i}^{\prime }\;d_{i}^{2}$$

where *n*_p_ is the number of residues collected in the space defined by the percentage of solvent accessibility *p* (%), and *d_i_* is the distance from the protein centroid to the centroid of residue *i*, in the space. Here, the protein centroid is the coordinates of its center of mass (CM). In this work, the space surface is dependent on *p* (%). When one selects a particular value of *P* (%), the number of residues *n*_p_, is collected within the space. When a value of *p* (%) increases, the volume of the space is also increased and the number of residues *n*_p_, residing in the space increases as well. The increment of *p* (%) is from 0 to 100%. Therefore, the spatial distribution of the second-order hydrophobic moment per residue, *H*_2_(d*_i_*), is represented as a function of *p* (%).

The Pearson correlation coefficient [36], R, was used to measure the correlation between the second-order moments as follows:

(11) $$R=\frac{\sum_{i}\left(x_{i}-\bar{x})(y_{i}-\bar{y}\right)}{\sqrt{\sum_{i}{\left(x_{i}-\bar{x}\right)}^{2}}\sqrt{\sum_{i}{\left(y_{i}-\bar{y}\right)}^{2}}}$$

where *x* denotes the second-order hydrophobic moment calculated with the *h_i_* selected from the consensus scale (Table 2), and *y* denotes the second-order hydrophobic moment calculated with the *h_i_* derived from the CHARMM partial atomic charges using Equation (8). These *x* and *y* were normalized by Equation (9). *x̄* and *ȳ* denote the mean of *x* and *y*, respectively. In this work, this equation is also used to test whether the correlation coefficient between two data sets is statistically significant to determine the equation of the regression line. The correlation coefficient calculated with Equation (11) is a linear correlation between the two data sets. If the correlation coefficient is close to 1, there is a very strong correlation between the two data sets.

## 3. Results and Discussion

### 3.1. Molecular Shape and Size

Fifteen protein structures (Table 1) in 150 nonhomologous protein chains were divided into three groups, depending on their asphericity (*δ*) as shown in Tables 3: Group I ranged from 1.49 × 10^−2^ to 2.48 × 10^−2^, Group II from 1.05 × 10^−1^ to 1.81 × 10^−1^, and Group III from 2.23 × 10^−1^ to 3.54 × 10^−1^. This division was also dependent on the number of residues (N), as shown in Table 1, to ensure a diverse range in molecular size. A suitable representation of molecular shape may be the gyration tensor, as shown in Equation (1). Diagonalization [36] of the gyration tensor gives three eigenvalues (*α*_1_, *α*_2_, and *α*_3_) as shown in columns 2, 3, and 4 of Table 3, respectively. Columns 5 and 6 of Table 3 show asphericity (*δ*) and the shape parameter (*S*), respectively. The values of *δ* were calculated with Equation (3), and those of *S* were calculated with Equation (4). The data show that the values of *δ* and of *S* for proteins in Group I are close to zero, suggesting that the shape of these proteins is close to a sphere. The shape of proteins can also be represented by semiaxes [32]. As shown in columns 7 and 8 of Table 3, the values of *a* and *b* in Group I are very close to each other, indicating that the molecular shape can also be identified as a sphere. As shown in Table 3, *α*_3_ is the largest eigenvalue for each protein structure in Groups II and III, compared with α_1_ and α_2_, so that the shape of proteins in Groups II and III can be concluded as a prolate ellipsoid [32]. Based on *δ* > 0 and *S* > 0, and *a* < *b*, one can also infer that the shape of the proteins in Groups II and III is a prolate ellipsoid.

**Figure 2:**
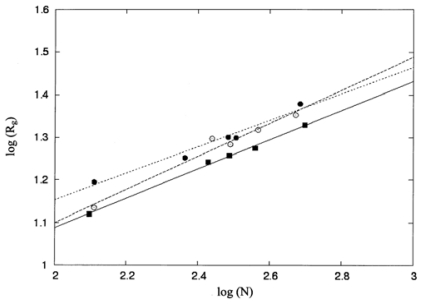
Plots of log (*R_g_*) *versus* log (*N*), where R*_g_* is the mean radius of gyration and N is the number of residues, for proteins in Group I (filled squares), Group II (open circles), and Group III (filled circles). The solid line (*R* = 0.99), the dashed line (*R* = 0.98), and the dotted line (*R* = 0.98) are the regression lines for proteins in Groups I, II, and III, respectively.

The last column in Table 3 lists the square roots of the mean square radius of gyration calculated according to Equation (6). The size of folded protein structures can be represented as the mean radius of gyration (***R****_g_*), which may follow the Flory scaling law [37–39], namely, *R_g_* ∝ *N*^ν^ (Å), where *N* is the number of amino-acid residues in a protein and *v* is a scaling exponent. As shown in Figure 2, the values of the mean radius of gyration (***R****_g_*) and the number of residues (*N*) were linearized by applying the Flory scaling law on a double logarithmic scale and were well fitted to linear lines (*y = mx + b*) by using the least-squares method. The Pearson correlation coefficient between each pair of parameters (log*N* and log*R_g_*) was calculated using Equation (11). The values for the correlation coefficient are 0.99, 0.98, and 0.98 for proteins in Group I, Group II, and Group III, respectively, indicating that the correlation between N and *R_g_* is highly significant. The values of *v*, obtained using the least-squares method, are 0.34, 0.39, and 0.32 for protein structures in Groups I, II, and III, respectively. These values are found to have a mean value of 0.35, which is close to 0.34 as predicted by the Flory scaling law for a polymer chain in a poor solvent [40–42]. Thus, these polypeptide chains may form a collapsed globule to minimize contact with the solvent molecules [42].

### 3.2. Distance Distribution Functions

**Figure 3:**
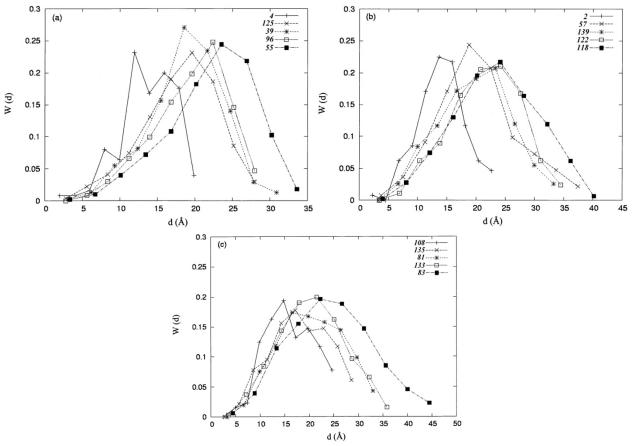
The distance distribution W (d) as a function of d (Å) for the protein structures, as shown in Table 1: (**a**) Proteins in Group I; (**b**) proteins in Group II; and (**c**) proteins in Group III. The indices, as shown in the legends of these figures, correspond to those shown in column 2 of Table 1.

The distances d (Å) from the protein centroid to the centroids of amino-acid residues in a protein, were grouped into 10 bins. The proportion of distances in each bin was plotted against d (Å) to obtain the distance distribution W (d), as shown in (Figures 3a, 3b, and 3c). Depicted in the figures are the results for protein structures in Groups I, II, and III, respectively. This scheme has been used to investigate the end-to-end distance distributions for poly(oxyethylene) chains [43]. The distance distributions may associate with the persistence length of a polymer chain described by the wormlike chain model [31]. The molecular size can also be characterized by the distance distributions. As shown in (Figures 3a, 3b, and 3c), these distributions all have a similar appearance, showing a single Gaussian-like distribution. These distributions, fluctuated about their most probable values, can be denoted as *d̄ ±* δ *d* (Å). A mean value, *d̄* (Å), which is associated with the mean radius of gyration, denotes the precise location of the most probable appearance of a distance distribution, and a value of δ*d* (Å) represents the breadth of its fluctuation. An increased extent of protein asphericity is correlated with increased fluctuations. For example, as shown in Table 1, proteins 1E70, 1JAE, and 1LAM all have a comparable number of residues. However, the fluctuations from their most probable values, as shown in Figures 3a, 3b, and 3c, have diverse values, such as 18.48 ± 9.65 Å, 22.10 ± 11.54 Å, and 24.46 ± 12.77 Å for proteins 1E70, 1JAE, and 1LAM, respectively.

### 3.3. The Second-Order Hydrophobic Moment

**Figure 4:**
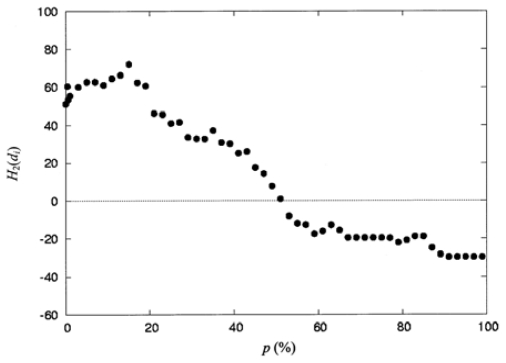
The spatial distribution of the second-order hydrophobic moment per residue, *H*_2_(d*_i_*), for the protein 3PYP, plotted as a function of the percentage of solvent accessibility, *p* (%). The second-order hydrophobic moment was calculated using *h_i_*′ selected from the consensus scales (Table 2).

The detailed results for the second-order moment per residue, calculated according to Equation (10) with hydrophobicity *h_i_′* selected from the consensus scale of Eisenberg (Table 2), are represented in Table 4 and Figure 4. Table 2 was normalized by Equation (9). Table 4 shows the details of the spatial distribution of the second-order hydrophobic moment per residue for protein 3PYP (see Table1) at a 2% resolution. The spatial distribution was calculated from the space collecting the residues that are completely buried (0%) to that collecting the residues that are completely exposed (100%) to the solvent. Figure 4 shows the distribution of the second-order hydrophobic moment per residue, *H*_2_(d*_i_*), as shown in Table 4, as a function of *p* (%); it is clear that the distribution transfers from the hydrophobic core with positive values of *H*_2_(d*_i_*) to the hydrophilic sites with negative values of *H*_2_(d*_i_*).

Table 5 shows the results of the Pearson correlation coefficient calculated using Eq. (11) for the correlation between two data sets of the second-order hydrophobic moments, calculated according to Equation (10) using two different sets of hydrophobic parameters: one is the hydrophobicity, *h_i_*′, selected from the consensus scale (Table 2) and the other is the *h_i_*′ calculated according to Equation (8) using the CHARMM partial atomic charges assigned to polar atoms with |*q_i_*| ≥ 0.25, 0.27, or 0.3. The both data sets were normalized by Equation (9). Comparing the values in each row of Table 5 clearly indicates that the values in the last column of Table 5 give the highest correlations, which were calculated with |*q_i_*| ≥ 0.3, for individual proteins. The mean values of correlation coefficient shown in the last column of Table 5 are 0.91 ± 0.02, 0.85 ± 0.03, and 0.86 ± 0.07 for proteins in Groups I, II, and III, respectively, showing that Group I with the lowest values of asphericity gives the strongest correlation.

**Figure 5:**
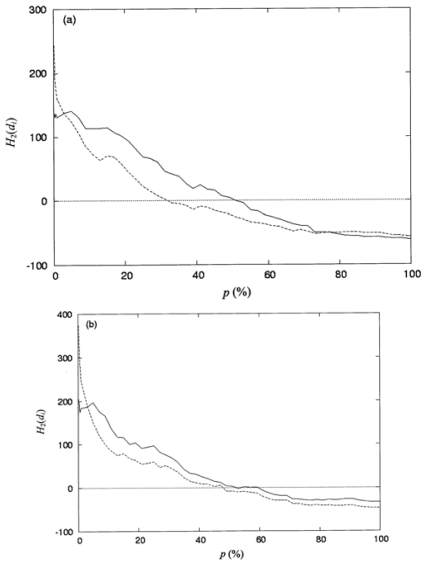

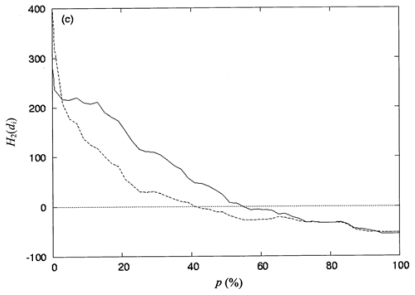
Comparative plots of the spatial distributions of the second-order hydrophobic moment per residue, *H*_2_(d*_i_*), *versus* the percentage of solvent accessibility, *p* (%), for (a) protein IE70, (b) protein 1JAE, and (c) protein 1LAM. The solid line and dashed line represent, respectively, the second-order hydrophobic moments calculated using *h_i_*′ selected from the consensus scales (Table 2) and the *h_i_*′ calculated according to Equation (8) with the CHARMM partial atomic charges, |*q_i_*| ≥ 0.3, assigned for polar atoms. The Pearson correlation coefficients between the spatial distributions of the second-order hydrophobic moments (solid and dashed lines) are 0.91, 0.91, and 0.90 for proteins IE70, 1JAE, and 1LAM, respectively.

Figures 5a, 5b, and 5c show the comparative plots for the spatial distributions of the second-order hydrophobic moment for protein structures IE70, 1JAE, and 1LAM, respectively, using Equations (10) and (11). Two data sets of second-order hydrophobic moments depicted in each of these figures were calculated using the hydrophobicity, *h_i_*′, selected from the consensus scale (Table 2) and the *h_i_*′ calculated according to Equation (8) using the CHARMM partial atomic charges assigned to polar atoms with |*q_i_*| ≥ 0.3, respectively. As shown in (Figures 5a, 5b, and 5c), the values of *h_i_*′ calculated using the CHARMM partial atomic charges overestimate the values of the second-order hydrophobic moments when *p* (%) is around zero. This is due to the fact that the denominator (*ASA_apo_* + *ASA_po_*) of Equation (8) yields small values at the hydrophobic core. In contrast, the other areas of *p* (%) give rise to a very strong Pearson correlation, having the values are 0.91, 0.91, and 0.90. Thus, *h_i_*′ calculated using the CHARMM partial atomic charges may provide an alternative way to assign the hydrophobicity for each amino-acid residue in a protein. Moreover, as shown in (Figures 5a, 5b, and 5c), the second-order hydrophobic moments give an overall shape of spatial distributions from the hydrophobic interior to the hydrophilic exterior. These results are in agreement with those of Silverman [4].

### 3.4. The Locations of the Spatial Profile of the Proteins at p_ (%) or at d̄_ (Å)

Table 6 shows the locations at which the spatial distribution of the second-order hydrophobic moments for the fifteen proteins vanishes and approaches a negative value. These locations are denoted as *p*_ (%) or *d̄*_ (Å) for the space defined by the percentage of solvent accessibility, *p* (%), or the average distance from the protein centroid to the centroids of the amino-acid residues in the same space, respectively. These locations show the transition from the hydrophobic core of the proteins to their hydrophilic exterior. Data listed in columns 2 and 3 of Table 6 are the values of the locations, *p*_ (%), calculated from the CHARMM partial atomic charges, |*q_i_*| ≥ 0.3, assigned for polar atoms, and from the consensus scale (Table 2), respectively, using the spaces defined by the percentage of solvent accessibility, *p* (%). The two data sets were normalized by Equation (9). Values in columns 4 and 5 of Table 6 are the average distances, *d̄*_ (Å), calculated using the same parameters of *h_i_*′ as those shown in columns 2 and 3 of Table 6, in the space corresponding to the percentage of solvent accessibility, *p*_ (%), shown for reference. As shown in Table 6, the values of *p*_ (%) in column 2 have an average value of 46.5 ± 7.9 %, whereas those of *p*_ (%) in column 3 have an average value of 57.9 ± 8.3 %. Thus, the hydrophobicity, *h_i_*′, calculated with the consensus scale yields a higher average value than that calculated with |*q_i_*| ≥ 0.3. The result of 46.5 ± 7.9 % may provide a criterion to determine whether residues in a protein are buried or exposed to a solvent. Gromiha *et al*. [14,15] have studied mutations in different solvent accessibilities and have considered the mutations that are exposed when the percentage of solvent accessibility, *p* (%), is greater than 50%.

## 4. Conclusions

A set of 150 nonhomologous protein chains has been randomly selected from PDBSELECT [26], which consisted of more than 4,000 protein chains. Fifteen protein structures have been selected from a set of 150 nonhomologous protein chains, based on the number of residues (*N*) and the extent of asphericity (*δ*), to ensure diversity of the molecular sizes and shapes. These fifteen protein structures have been the subject of a comparative study using second-order hydrophobic moments. This may provide an alternative approach to the assignment of hydrophobicity to each amino-acid residue in the proteins by using the CHARMM partial atomic charges. The Pearson correlation coefficient for each protein structure has been used to compare the spatial distributions of second-order hydrophobic moments calculated using the hydrophobicity, *h_i_*, obtained from the consensus scale of Eisenberg [5] with that calculated using Equation (8) for the *h_i_* derived from CHARMM partial atomic charges assigned to atoms in a protein. These two data sets were normalized by Equation (9). The Pearson correlation coefficient between the two data sets shows a strong correlation for each of these fifteen protein structures when the absolute values of the CHARMM partial atomic charges are greater than 0.3, assigned for polar atoms. These comparative results suggest that the hydrophobicity of each type of amino-acid residue in the proteins can probably be obtained using the CHARMM partial charges assigned to atoms in amino-acid residues. The spatial distributions of the second-order hydrophobic moment have the overall shape of the transition from hydrophobic interior to hydrophilic exterior. The transition at a position where the value of the second-order hydrophobic moment vanishes and approaches a negative value was denoted as *p*_ (%), and the average value of *p*_ (%) is 46.5 ± 7.9%, which has been calculated with CHARMM partial atomic charges, |*q_i_*| ≥ 0.3, assigned for polar atoms. This may give the value of the transition point from the buried sites to exposed sites for amino-acid residues in the protein structures.

## Figures and Tables

**Table 1:** List of proteins used in this work.

Protein	Protein index	PDB code	No. residues
I			
Photoactive yellow protein	4	3PYP	125
Exonuclease III	125	1AKO	268
Carboxypeptidase A	39	2CTC	307
Cellulase cela	96	1CEM	363
Myrosinase	55	1E70	499
II			
Lysozyme	2	3LTZ	129
Dienoyl-coa isomerase	57	1DCIA	275
Sulfate-Binding protein	139	1SBP	309
Maltodextrin-binding protein	122	3MBP	370
Alpha-amylase	118	1JAE	470
III			
Cytochrome C′	108	1CPQ	129
Molybdate transport protein	135	1AMF	231
L-arabinose-binding protein	81	1ABE	305
Phosphate-binding protein	133	2ABH	321
Leucine aminopeptidase	83	1LAM	484

**Table 2:** Values of ASA (Å^2^) and the consensus scale for each amino-acid residue.

Residue	ASA a	Consensus scale (*h_i_*) b
Ala	116.5	0.25
Arg	249.7	−1.80
Asn	163.2	−0.64
Asp	156.2	−0.72
Cys	140.2	0.04
Gln	188.9	−0.69
Glu	183.8	−0.62
Gly	84.9	0.16
His	204.3	−0.40
Ile	183.6	0.73
Leu	193.5	0.53
Lys	211.3	−1.10
Met	205.9	0.26
Phe	223.7	0.61
Pro	148.3	−0.07
Ser	124.8	−0.26
Thr	144.1	−0.18
Trp	266.3	0.37
Tyr	236.0	0.02
Val	158.0	0.54

a Solvent-accessible surface area (ASA) of each residue type X in the tripeptide (Gly-X-Gly) at the extended state, calculated using ASC [34];

b The hydrophobicity consensus scale of Eisenburg [5].

**Table 3:** The results of molecular shape and size.

PDB code	*α*_1_^a^	*α*_2_^a^	*α*_3_^a^	0^2^ ×*δ*^b^	10^3^× *S*^c^	*a*^d^	*b*^d^	*R_g_*^e^
I								
3PYP	41.11	61.82	71.06	2.33	−4.33	16.04	18.85	13.19
1AKO	78.40	96.64	129.61	2.18	3.05	20.92	25.46	17.45
2CTC	84.32	100.38	141.79	2.48	5.30	21.49	26.63	18.07
1CEM	93.46	116.77	143.17	1.49	0.39	22.93	26.76	18.80
1E70	110.18	169.06	176.62	1.91	−5.02	26.42	29.72	21.35
II								
3LTZ	35.84	48.75	101.55	10.49	57.61	14.54	22.53	13.64
1DCIA	72.74	96.28	223.74	12.84	83.38	20.56	33.45	19.82
1SBP	56.12	105.62	206.44	12.79	51.45	20.17	32.13	19.21
3MBP	74.47	115.72	242.46	12.28	65.10	21.81	34.82	20.80
1JAE	88.54	105.91	312.60	18.13	151.02	22.05	39.53	22.52
III								
1CPQ	26.97	39.53	178.83	35.41	410.89	12.89	29.90	15.66
1AMF	35.21	75.05	208.33	24.30	188.55	16.60	32.27	17.85
1ABE	53.21	89.04	256.31	22.25	183.73	18.86	35.80	19.96
2ABH	65.13	68.09	262.66	24.53	242.78	18.25	36.24	19.90
1LAM	44.08	141.19	388.73	28.90	211.84	21.46	44.09	23.94

a The eigenvalues of gyration tensor, obtained by diagonalization of Equation (1). The values were sorted according to increasing magnitude;

b The asphericity (*δ*), calculated according to Equation (3);

c The shape parameter (S), calculated according to Equation (4);

d Semiaxes (in Å) [32], calculated with *a* = [5(*α*_1_ +*α*_2_)/2]^1/2^ and *b* = (5*α*_3_)^1/2^;

e The mean radius of gyrations (in Å), calculated according Equation (6).

**Table 4:** The details of the spatial distribution of the second-order moment for 3PYP.

*P* (%)	*n_p_*^a^	*H*_2_(d*_i_*) b	*P* (%)	*n_p_*^a^	*H*_2_(d*_i_*) b
0.00	13	51.14	47.0	88	14.18
0.40	17	60.63	49.0	90	7.79
0.60	18	53.46	51.0	92	1.01
1.00	21	55.51	53.0	98	−8.13
3.00	24	60.15	55.0	99	−11.95
5.00	30	62.87	57.0	102	−12.64
7.00	30	62.87	59.0	103	−17.51
9.00	33	61.17	61.0	104	−16.08
11.0	39	64.58	63.0	106	−12.87
13.0	42	66.37	65.0	109	−15.63
15.0	44	72.18	67.0	111	−19.49
17.0	48	62.50	69.0	111	−19.49
19.0	50	60.74	71.0	114	−19.64
21.0	54	46.35	73.0	114	−19.64
23.0	57	45.69	75.0	114	−19.64
25.0	63	41.04	77.0	117	−19.73
27.0	64	41.57	79.0	119	−22.16
29.0	66	33.47	81.0	120	−20.88
31.0	68	32.77	83.0	121	−18.87
33.0	70	32.65	85.0	121	−18.87
35.0	73	37.21	87.0	123	−24.80
37.0	76	30.78	89.0	124	−28.33
39.0	77	30.14	91.0	125	−29.56
41.0	81	25.23	93.0	125	−29.56
43.0	82	26.02	95.0	125	−29.56
45.0	86	17.47	97.0	125	−29.56

a Number of residues collected in the space defined by *p* (%);

b The second-order hydrophobic moment per residue, calculated according Equation (10).

**Table 5:** The Pearson correlation coefficient between two data sets: the spatial distribution of the second-order hydrophobic moment calculated with the consensus scale and that calculated with Equation (8) derived from the CHARMM partial atomic charges.

PDB code	Correlation coefficient (R) a		
	|*q_i_*| ≥ 0.25 b	|*q_i_*|≥ 0.27 c	|*q_i_*| ≥ 0.3 d
I			
3PYP	0.89	0.89	0.94
1AKO	0.78	0.77	0.87
2CTC	0.86	0.87	0.91
1CEM	0.85	0.86	0.92
1E70	0.88	0.87	0.91
II			
3LTZ	0.63	0.63	0.85
1DCIA	0.78	0.79	0.82
1SBP	0.70	0.70	0.86
3MBP	0.59	0.59	0.82
1JAE	0.86	0.87	0.91
III			
1CPQ	0.81	0.81	0.96
1AMF	0.70	0.70	0.88
1ABE	0.72	0.74	0.81
2ABH	0.62	0.62	0.77
1LAM	0.87	0.87	0.90

a The Pearson correlation coefficient, calculated according to Equation (11);

b The values of the coefficient calculated using the CHARMM partial atomic charges, |*q_i_*| ≥ 0.25, assigned for polar atoms in the protein structures;

c The values of the coefficient calculated using the CHARMM partial atomic charges, |*q_i_*| ≥ 0.27, assigned for polar atoms in the protein structures;

d The values of the coefficient calculated using the CHARMM partial atomic charges, |*q_i_*| ≥ 0.3, assigned for polar atoms in the protein structures.

**Table 6:** The results of the spatial distributions of the second-order hydrophobic moment at *p*_ (%) or at *d̄*_ (Å).

**PDB code**	*p*_(%)^a^		*d̄* _ **(Å)**^b^	
		
	|*q_i_*| ≥ 0.3 c	**C scale**^d^	|*q_i_*| ≥ 0.3 e	**C scale**^f^
I				
3PYP	49	53	11.45	11.79
1AKO	59	53	15.79	15.45
2CTC	47	49	16.14	16.24
1CEM	41	49	16.80	17.10
1E70	33	51	18.52	19.57
II				
3LTZ	39	55	11.56	12.16
1DCIA	49	57	16.66	17.33
1SBP	61	61	17.43	17.43
3MBP	51	61	18.72	19.07
1JAE	49	53	20.56	20.65
III				
1CPQ	53	81	13.92	14.70
1AMF	49	59	15.49	15.77
1ABE	41	71	17.23	18.39
2ABH	37	61	17.00	17.95
1LAM	43	55	20.67	21.20

a The space defined by the percentage of solvent accessibility, *p*_(%), at which the spatial distribution of the second-order moment vanishes and approaches a negative value;

b The average distance from the protein centroid to the centroids of the amino-acid residues in the same space defined by (%) *p*_ as shown in footnote (a);

c The values of *p*_(%), calculated using *h_i_*′ [Equation (9)] derived from the CHARMM partial atomic charges, |*q_i_*| ≥ 0.3, assigned for polar atoms in the protein structures.

d The values of *p*_ (%), calculated using the consensus scales.

e The values of *d̄*_ (Å), calculated using *h_i_*′ [ Equation (9)] derived from the CHARMM partial atomic charges, |*q_i_*| ≥ 0.3, assigned for polar atoms in the protein structures.

f The values of *d̄*_ (Å), calculated using the consensus scales.
